# Rapid trait evolution drives increased speed and variance in experimental range expansions

**DOI:** 10.1038/ncomms14303

**Published:** 2017-01-27

**Authors:** Christopher Weiss-Lehman, Ruth A Hufbauer, Brett A Melbourne

**Affiliations:** 1 Department of Ecology and Evolutionary Biology, University of Colorado, Boulder, Colorado 80309, USA; 2 Biofrontiers Institute, University of Colorado, Boulder, Colorado 80309, USA; 3 Department of Bioagricultural Sciences and Pest Management, Colorado State University, Fort Collins, Colorado 80523-1177, USA; 4 Graduate Degree Program in Ecology, Colorado State University, Fort Collins, Colorado 80523, USA; 5 UMR Centre de Biologie et Gestion des Populations, INRA, 34988 Montferrier sur Lez, France

## Abstract

Range expansions are central to two ecological issues reshaping patterns of global biodiversity: biological invasions and climate change. Traditional theory considers range expansion as the outcome of the demographic processes of birth, death and dispersal, while ignoring the evolutionary implications of such processes. Recent research suggests evolution could also play a critical role in determining expansion speed but controlled experiments are lacking. Here we use flour beetles (*Tribolium castaneum*) to show experimentally that mean expansion speed and stochastic variation in speed are both increased by rapid evolution of traits at the expansion edge. We find that higher dispersal ability and lower intrinsic growth rates evolve at the expansion edge compared with spatially nonevolving controls. Furthermore, evolution of these traits is variable, leading to enhanced variance in speed among replicate population expansions. Our results demonstrate that evolutionary processes must be considered alongside demographic ones to better understand and predict range expansions.

## Abstract (web summary)

Spatial structure provides unique opportunities for evolution during range expansions. Here, the authors show experimentally using the red flour beetle, *Tribolium castaneum*, that dispersal and growth can evolve through spatial processes, increasing expansion speed and its variance.

Evolution is predicted to change the dynamics of range expansions through multiple processes. For example, the expanding population may encounter different biotic and abiotic conditions in the newly colonized habitat that impose novel selection pressures^1^. Evolution in response to a novel environment is necessarily context dependent and, although important, difficult to generalize across range expansions. However, three other processes by which evolution can change the dynamics of range expansions are direct outcomes of the intrinsic spatial population structure formed during range expansion and should therefore be general to any range expansion. First, spatial sorting^2^, the nonrandom aggregation of highly successful colonizers at the expansion edge, and subsequent assortative mating among them, can lead to increases in traits related to colonization (such as dispersal ability)^2^,^3^,^4^. Second, the selection imposed by population density varies across the expanding range, with individuals at the expansion edge typically experiencing lower density than individuals in the core of a species' range^5^,^6^. Traits conferring fitness at high densities, generally referred to as competitive ability, are expected to evolve upwards in the core of the range but not at the edge where densities are low^6^,^7^. In contrast, edge populations are expected to evolve higher fecundity at the expense of competitive ability^6^, yielding higher intrinsic growth rates when compared with the core. Here, intrinsic growth rate refers to the population growth rate achievable in a given environment unhindered by any negative influence of population density and is typically determined by fitness at low density when competition is absent. A third way that spatial evolution can change the dynamics of range expansions is that allele frequencies can be influenced by the small population sizes and repeated founder events associated with the edge of the expanding population, resulting in the recently discovered phenomenon of gene surfing^8^,^9^,^10^,^11^,^12^,^13^,^14^,^15^. In gene surfing, serial founding events at the expansion edge^16^ can allow deleterious alleles to increase in frequency and travel with the expansion edge, whereas advantageous alleles can be lost because of chance events^9^,^14^. Increased frequency of deleterious alleles in edge populations is predicted to reduce mean fitness^9^,^14^ and thus decrease intrinsic growth rates at the edge, regardless of density. These three spatial evolutionary processes could act alone or together to change the speed of a range expansion over time.

Expansion speed is determined largely by growth rate and dispersal at the edge^17^,^18^, and hence the effect of evolution on expansion speed should depend on the balance of evolutionary processes that increase or decrease growth rate and/or dispersal at low density. Theory shows that evolved increases in intrinsic growth rate or dispersal ability at the edge should increase expansion speed^4^,^6^,^19^,^20^. On the other hand, theory shows that gene surfing should decrease fitness at the expansion edge^9^,^14^, thus depressing intrinsic growth rates and slowing expansion^8^. Given the stochastic nature of evolutionary processes, the evolutionary outcome of any one realization of a range expansion will be partly randomly determined and is thus likely to be unique. Indeed, the stochastic and potentially opposing effects of different spatial evolutionary processes on expansion speed offer a possible explanation for previous experimental work showing that demographic factors alone are not sufficient to explain observed variability among range expansions^5^. A theoretical model combining dispersal evolution with gene surfing predicts that evolution will increase stochasticity in expansion speed among range expansions relative to models of expansion that do not include evolutionary processes^19^. However, evolution of increased dispersal is still predicted to dominate, so that evolution is predicted to lead overall to an increase in mean expansion speed along with increased variance^19^.

The three spatial evolutionary processes outlined above (spatial sorting, selection by density and gene surfing) have varying degrees of empirical support. For example, evolved increases in dispersal ability have been observed in natural and experimental range expansions^21^,^22^,^23^,^24^,^25^ as have signatures of gene surfing^10^,^26^,^27^. On the other hand, investigation of spatial evolution of competitive ability via selection across the density gradient of a range expansion has been primarily theoretical^6^,^7^. Implicit in each of the three spatial evolutionary processes is the idea that the dynamic development of spatial structure across the species range, from core to edge, drives evolution in range expansions. This has yet to be tested empirically, and controlled experiments are needed to determine the effects of spatial structure on evolutionary dynamics of range expansions. Here, we evaluate the role played by spatial structure in driving evolution in experimental range expansions using laboratory microcosms of the red flour beetle, *Tribolium castaneum*.

We founded replicate experimental populations from the same large, well-mixed source population, and allowed them to expand from the founding point. To isolate the effects of evolution due to spatial structure on expansion speed and variance, we compared expanding populations subjected to two experimental treatments. In one treatment, populations were allowed to develop natural spatial genetic structure over time (that is, spatial evolution was allowed), and in the other treatment we prevented the development of spatial genetic structure (that is, spatial evolution was prevented) by randomly shuffling the location of individuals without disrupting population density or demographic processes (Methods). After eight generations of range expansion, we compared the effect of the treatments on two key traits that contribute to expansion speed, dispersal ability and intrinsic growth rate by assaying G1 (first generation) descendants of beetles from structured and shuffled populations (Methods).

The treatment allowing spatial evolution has a significantly higher mean spread rate compared with the shuffled treatment as well as heightened variability in spread rates. The trait experiments suggest that these patterns are driven by evolution of dispersal and growth rate in beetles at the expansion edge of structured populations. Our results demonstrate the importance of spatial evolutionary changes in determining the dynamics of range expansion over short timescales.

## Results

### Speed and variance of range expansion

**Figure 1:**
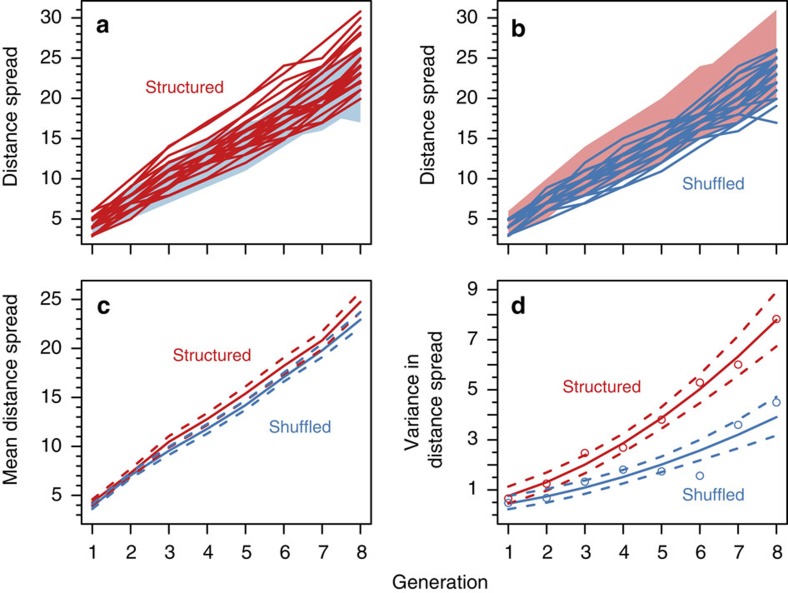
Speed and variability of structured and shuffled range expansions. Experimental results of range expansions with structured (*n*=28) and shuffled (*n*=29) populations initiated from a single well-mixed source population. (**a**,**b**) Distances spread in each treatment. The lines show data for individual replicates whereas the shaded regions show the observed range of distance spread in the other treatment for reference. (**c**) Mean distance spread through time for each treatment (solid lines) and sample estimated 95% confidence intervals (dashed lines). (**d**) Model-estimated variances for each treatment with 95% confidence intervals. The observed variances for each treatment are shown as points. In all panels the structured treatment is shown in red and the shuffled treatment in blue. Spatial evolution resulted in a higher mean expansion speed (parametric bootstrap, treatment by generation interaction: *P*=0.0137) and higher variance in expansion speeds (likelihood ratio test, treatment by generation interaction: *P*=1.93 × 10^−5^).

Spatial evolution led to a 6% (95% confidence interval (CI) 1.3–11.6%) higher mean expansion speed compared with populations in which individuals (and thus alleles) were shuffled each generation (Fig. 1; parametric bootstrap, treatment by generation interaction: *P*=0.0137). Importantly, variance in distance spread among replicate populations was increased by spatial evolutionary processes (Fig. 1; likelihood ratio test, treatment by generation interaction: *P*=1.93 × 10^−5^), leading to almost doubled variance in the distance spread of structured compared with shuffled landscapes by the eighth generation. Furthermore, to test whether the increased variance in speed was only a result of the increased mean, we analysed the coefficient of variation (CV) and the variance to mean relationship and confirmed that spatial evolution resulted in higher variation in expansion speeds independent of the differences in mean expansion speed (Supplementary Fig. 1; CV, likelihood ratio test, treatment effect: *P*=0.004; variance to mean relationship, treatment–mean interaction, *P*=0.025).

By founding each landscape with randomly selected beetles from the same well-mixed source population, we expected differences between treatments to be negligible at first, before the shuffle treatment began or had an effect, and to develop over time. This expectation was confirmed, as distance spread (Fig. 1c; Poisson generalized linear mixed model, generation 1, *P*=0.41, generation 2, *P*=0.72) and variance (Fig. 1d; *F*-test, generation 1, *P*=0.41, generation 2, *P*=0.11) did not differ significantly by treatment in the first two generations. Two of the structured landscapes, however, spread further than any shuffled landscapes in the first generation (Fig. 1a). To exclude the possibility of a fortuitous random draw, we redid the analyses excluding these two landscapes and the results showed the same patterns (expansion speed, treatment by generation interaction: *P*=0.0138; variance, treatment by generation interaction: *P*=0.0003; CV, treatment effect after G1: *P*=0.002; variance to mean relationship, treatment–mean interaction: *P*=0.030).

### Trait evolution

**Figure 2:**
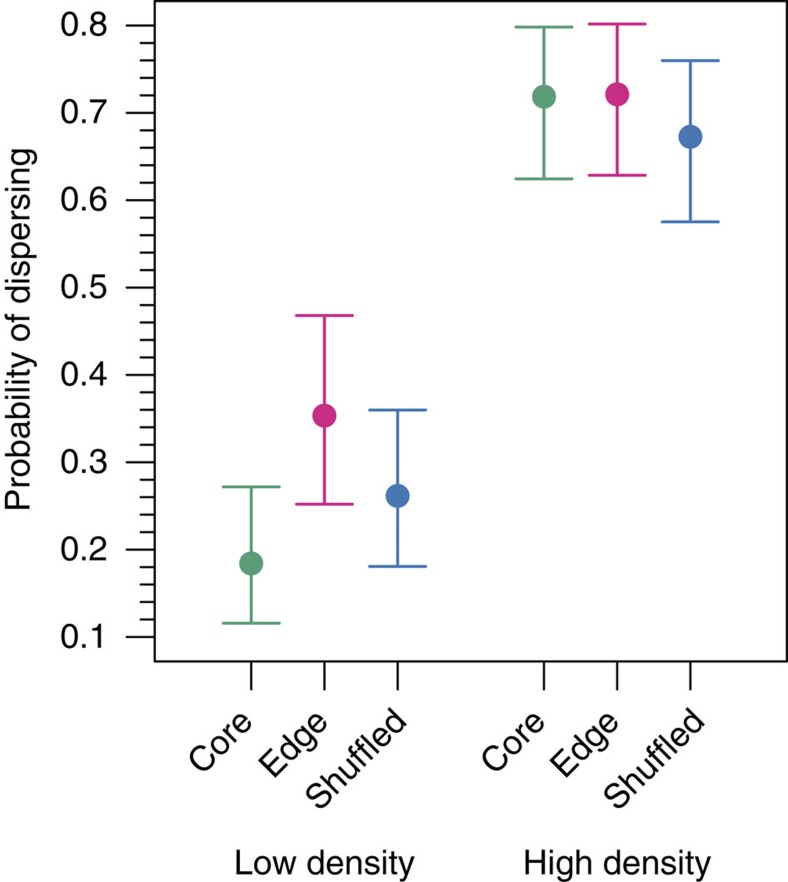
Evolved density dependence of dispersal probability. Mean probability of beetles dispersing out of their patch of origin with low and high initial densities (10 and 40 adult beetles). Results for G1 descendants from the core (*n*=28) and edge (*n*=28) of structured landscapes and G1 descendants from shuffled landscapes (*n*=58) are shown. Filled circles are model estimates from a binomial generalized linear mixed effects model with a logit link function. Error bars are bootstrapped 95% confidence intervals. Core populations are shown in green, edge populations in fuchsia and shuffled populations in blue. Spatial evolution led to a higher proportion of beetles dispersing from edge populations at low density (parametric bootstrap, interaction of density and location: *P*=0.0008).

After eight generations of range expansion, G1 descendants from the expansion edge of structured populations had higher dispersal under low-density conditions compared with G1 descendants from the range core or shuffled populations (Fig. 2; parametric bootstrap, interaction of density and location: *P*=0.0008). Dispersal was 92% (CI 42–172%) higher at the edge than the core and 35% (CI −0.3 to 83%) higher at the edge than in shuffled populations. Under high-density conditions, the dispersal tendencies of G1 descendants from all populations were similar (Fig. 2 and Supplementary Fig. 2).

**Figure 3:**
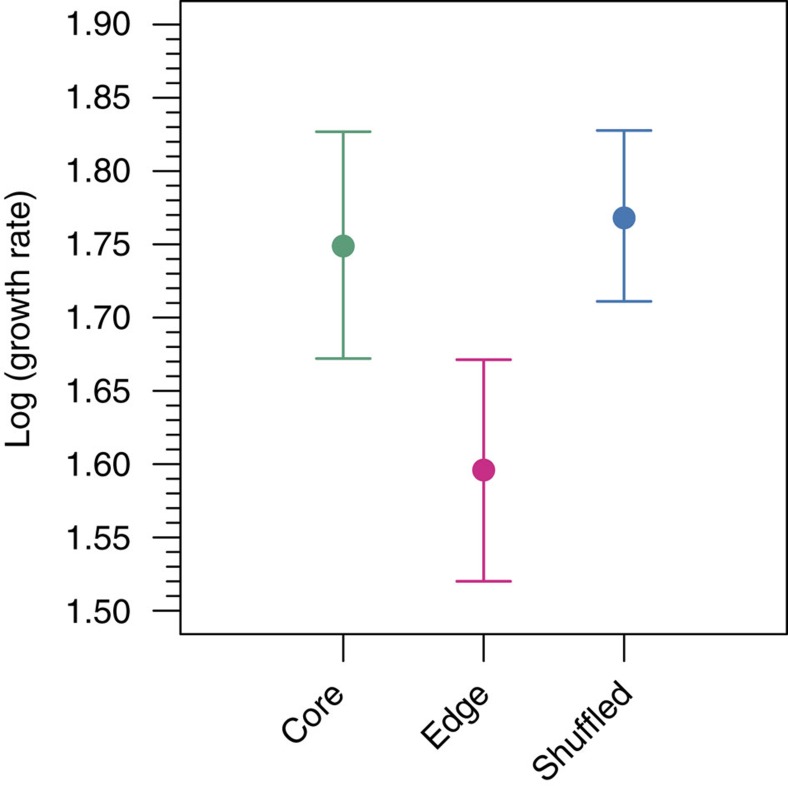
Evolved intrinsic growth rates among spatial locations and shuffled populations. The mean natural log of growth rate (defined as the ratio of population density at time *t*+1 to population density at time *t*) for G1 descendants of beetles taken from the core and edge of structured landscapes and G1 descendants from shuffled landscapes. As there was no significant difference in density dependence of growth rates among core, edge and shuffled beetles, filled circles represent model estimates averaged over density levels from a linear mixed effects model and provide a relative measure of intrinsic growth rate. The numbers of samples at each of the four density levels respectively were: core, *n*=20, 24, 22 and 22; edge, *n*=23, 27, 23 and 23; and shuffled, *n*=38, 48, 39 and 39. Sample sizes differ among locations and densities because of variation in the number of G1 offspring available. Error bars are bootstrapped 95% confidence intervals.

Intrinsic growth rates were lower among G1 descendants from the expansion edge of structured populations when compared with G1 descendants from range cores and shuffled landscapes (Fig. 3; parametric bootstrap, effect of location: *P*=0.0012). Intrinsic growth rate was 9.9% (CI 4.7–14.9%) lower at the edge compared with shuffled populations, whereas core and shuffled populations were similar. The degree to which growth rate was lower for edge populations was similar across densities and not significantly related to density (Supplementary Fig. 3; parametric bootstrap, interaction of density and location: *P*=0.4285).

## Discussion

By replicating experimental range expansions and comparing them with nonstructured controls, we can evaluate rigorously the role of spatial evolutionary processes in range expansions. The congruence of our results with theoretical studies^4^,^19^,^28^ confirms that spatial evolutionary processes can explain previously reported patterns of increased mean and variance in expansion speeds^5^,^29^. Additionally, our findings are consistent with the work of Ochocki and Miller (ref. 30) in this issue, wherein a remarkably similar approach with a different model system was used to examine the effects of spatial structure on range expansion. Over eight generations, we found a small increase in mean speed and a larger increase in variance in spatially evolving populations compared with populations that were not evolving spatial structure. Furthermore, we observed rapid differential evolution, within eight generations, of two key traits, dispersal ability and intrinsic growth rate, in edge versus core populations of spatially evolving populations. This rapid spatial evolution of traits likely explains the increased speed and variance of spatially evolving populations compared with controls.

Within structured populations, higher low-density dispersal rates and lower intrinsic growth rates evolved at the edge compared with the core and with shuffled populations, leading overall to a slight increase in mean expansion speed relative to shuffled populations. Higher low-density dispersal rates at the edge (where density is low) should lead to higher expansion speeds, whereas lower intrinsic growth rates at the edge should lead to a decrease in expansion speed because fewer colonists are produced. The realized expansion speed is thus a balance of these rates and the increase in mean expansion speed observed suggests that on average the positive effect of dispersal evolution outweighed the negative effect of growth rate evolution. An order of magnitude approximation of expected speed based on our experimental measurements of dispersal and intrinsic growth rate can be calculated using 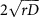, where *r* is the instantaneous intrinsic growth rate and *D* the diffusion coefficient (Methods). This formula applies across a wide range of expansion models^18^, and applying it to our data gives a maximum increase in speed of 13% for structured compared with shuffled populations, well in line with the observed 6% increase. The observed increase in speed is expected to be lower than the maximum for two reasons. First, beetles from the higher-density patches just behind the edge do contribute to spread but should not experience the same boost in dispersal as edge beetles, given the density dependence of the boost (Fig. 2). Second, the approximated maximum relies upon dispersal and intrinsic growth measured when they presumably differed the most, at the end of the experiment, and does not account for values of these traits diverging over time.

Our results support the theoretical prediction that stochasticity in the evolution of dispersal ability and intrinsic growth rate at the expanding edge combine to increase variation in expansion speed^19^. Although on average individuals from the expanding edge displayed heightened dispersal ability and decreased growth rates compared with individuals from the core and shuffled populations, specific trait values were highly variable (Supplementary Figs 2 and 3) and uncorrelated with each other (Supplementary Fig. 4). Thus, as predicted^19^, stochastic combinations of evolving traits at the expansion edge, such as slower dispersers with low intrinsic growth rates or faster dispersers with higher intrinsic growth rates, would increase variance in expansion speed.

Patterns of traits after eight generations provide some clues to the evolutionary processes that might be dominant across the range expansions. For dispersal, as the low-density dispersal tendencies of G1 descendants in structured populations were within the observed variation of shuffled populations (Supplementary Fig. 2), the difference between edge and core in structured populations was likely because of selection on standing genetic variation of high dispersing genotypes at the edge and low dispersing genotypes in the core, rather than evolution of new genotypes via mutation, consistent with the theory of spatial sorting^2^. For intrinsic growth rate, spatial sorting is expected to lead to higher growth rate at the expanding edge because producing more offspring increases colonization ability^31^. However, we observed lower intrinsic growth rates in edge populations, suggesting that spatial sorting is not the dominant process driving the evolution of growth rates across the landscapes. Similarly, if selection by density dominated, we would expect evidence of an evolved tradeoff across densities between the expansion edge and range core that we did not find. One possible explanation is that individuals from the leading edge evolved reduced intrinsic growth rates because of a tradeoff between dispersal and fecundity, such that highly dispersive individuals at the edge evolved lower fecundity because of greater energy expenditures on movement or metabolism^6^,^32^. However, the correlation between dispersal ability and intrinsic growth rate in G1 descendants from edge individuals was low, providing little evidence for a tradeoff (Supplementary Fig. 4; Pearson's *r*=0.0785). In contrast, evolution of reduced intrinsic growth rates in individuals from the edge is consistent with predictions of gene surfing in range expansions^8^,^9^,^14^, where the accumulation of deleterious alleles can be expected to reduce intrinsic growth rates, regardless of density. It is feasible that gene surfing of deleterious alleles could have developed quickly, as this process is predicted to be particularly potent in systems with relatively low carrying capacities (∼250 individuals per patch in our system) and high genetic load such as ours^33^ because of the greater influence of genetic drift, founder effects and inbreeding at the edge in such systems^9^. Future work should focus on the genetic basis of trait evolution to test these hypotheses.

By removing the possibility for spatial evolution in the shuffled landscapes, we obtained an estimate of its relative effects. The differences in traits among core, edge and shuffled populations (each initiated randomly from the same well-mixed stock populations and thus starting with highly similar trait distributions) measured under common controlled conditions demonstrate that rapid evolution occurred and in what relative direction. Namely, edges evolved higher dispersal and lower intrinsic growth rate compared with shuffled and core. Because we cannot determine the direction of evolution absolutely, a possibility worth considering is that shuffled populations and populations in the core of the structured landscapes both evolved reduced dispersal ability and increased intrinsic growth rate compared with edge populations rather than, or in addition to, edge populations evolving increased dispersal ability or reduced growth rate. Two observations suggest that this direction of evolution is unlikely. First, the observed trait patterns are consistent with proposed mechanisms for evolution at the edge. For dispersal, the pattern of higher dispersal at the edge, lower dispersal in the core and shuffled populations intermediate is consistent with spatial sorting. Similarly, the pattern of lower intrinsic growth rate at the edge and matching higher intrinsic growth rates in core and shuffled populations is consistent with gene surfing. Second, if evolution were primarily in the core and shuffled treatments, this would imply selection for lower dispersal and higher intrinsic growth rates across the landscape, and the inability of edge populations in structured landscapes to respond to that selection. Mechanisms for a lack of response to selection at the edge are less plausible and would, in any case, need to involve evolutionary processes such as genetic drift in the small edge populations or gene surfing constraining an adaptive response. Thus, the relative shifts in dispersal and intrinsic growth rate are in accord with theories for evolutionary processes driving these crucial traits along the expanding edge, regardless of the absolute direction of trait evolution.

Rapid evolution of dispersal at the edge provides a direct explanation for increasing expansion speeds observed in range expansions^29^. Furthermore, evolution of heightened dispersal ability at the edge compared with the core is countered by opposing evolutionary decreases in intrinsic growth rate of edge individuals compared with the core, and these stochastic processes combine to generate variance in expansion speed. It is thus critical to understand the role of spatial evolutionary processes in range expansions to predict accurately the scope of possible outcomes as a species spreads^5^,^19^. Building explicit consideration of spatial and temporal evolution into models for the prediction of spatial spread of invasive species or range shifts induced by climate change, most of which currently rely on purely demographic processes, will be a fruitful research area^34^. In particular, our experimental results suggest that the inclusion of spatial evolutionary processes in such models will provide substantial gains in their predictive accuracy and estimates of uncertainty.

## Methods

### Experimental system

We used *T. castaneum*, the red flour beetle, in experimental microcosms to test the effects of spatial evolutionary mechanisms on range expansions. *T. castaneum* populations were kept in 4 cm by 4 cm by 6 cm acrylic containers with 20 g of a standard medium (95% wheat flour and 5% brewer's yeast). We will henceforth refer to a container complete with medium as a patch. Patches can contain single, isolated populations of *T. castaneum* (as used in growth assays) or be connected in linear arrays via 2 mm holes drilled in the sides to form landscapes of patches^5^ linked by dispersal (as used in the range expansion experiment and dispersal assays). The life cycle of *T. castaneum* was constrained to mimic that of a seasonally breeding organism with non-overlapping generations and a discrete dispersal phase^35^, a life history found commonly among plants and animals. Adult beetles were placed in individual patches with fresh standard medium for 24 h to reproduce and start the next generation. At the end of the reproduction phase, adults were removed from the patches and the eggs were left to mature into adults over a 34-day period. In landscapes linked by dispersal via holes drilled in the sides of the patches, thin plastic sheets were placed between patches for 34 days to prevent dispersal. These sheets were removed for 24 h on day 34 to allow for a discrete dispersal phase before the populations were censused and adult beetles were placed in fresh medium to begin the next generation. Although egg deposition was constricted to only this 24 h period, mating could occur during that 24 h period and at any point prior, once beetles reached maturity (usually a few days before the dispersal period). Therefore, a single beetle dispersing to an unoccupied patch could give rise to offspring in the next generation if it was a mated female. This experimental protocol yielded a 35-day generation time. Beetles were kept in incubators at 31 °C with ∼80% relative humidity. Three incubators were used and landscapes or single isolated patches were randomized among and within incubators once each week to prevent systematic effects of incubation conditions.

### Range expansion experiment

We tested the effects of spatial evolutionary processes on range expansions with 60 experimental landscapes of *T. castaneum* divided between two treatments that we will call ‘structured' and ‘shuffled'. In the structured treatment, to begin each generation we returned beetles to the same patch in which we recorded them, thus allowing the formation of spatial genetic structure because of evolutionary processes. In the shuffled treatment, we prevented spatial evolution by randomizing the spatial location of beetles within landscapes at the start of each generation. To randomize beetles, we first recorded population densities in each patch after dispersal, and then all beetles from a landscape were mixed together and redistributed throughout the landscape according to the recorded density of each patch. This procedure disrupted the formation of spatial genetic structure by decoupling an individual's genetics from its location, but maintained the demographic processes of the range expansion (for example, density-dependent growth or dispersal). Landscapes from each treatment were additionally divided randomly among three temporal blocks.

Following common procedures from other experimental evolution studies^36^,^37^,^38^,^39^,^40^,^41^,^42^,^43^,^44^,^45^, populations were initially founded from the same large, well-mixed population and then randomly assigned to an experimental treatment (structured or shuffled). Landscapes were founded by placing 20 randomly selected adult beetles into the first patch of the landscape for 24 h to reproduce. Expansions proceeded for eight generations. Both treatments began with 30 replicate landscapes but several replicates were lost because of laboratory mishaps, yielding 28 replicates of the structured treatment and 29 replicates of the shuffled treatment.

### Testing for spatial trait evolution

At the end of the eighth generation of expansion, we tested for evolved differences in dispersal ability and growth rate. For each structured landscape, we drew 20 beetles at random from the range core (that is, the first patch of the landscape) and took the 20 furthest forward at the range edge (drawing randomly where necessary to make up to 20). For each shuffled landscape, we drew two random samples of 20 beetles each (as random beetles began the generation at the core and edge of shuffled landscapes). Each sample of 20 beetles was placed in an individual patch to reproduce for 24 h. We used the first-generation offspring (that is, G1 generation) to perform assays of dispersal ability and growth rate within a common environment to determine if there were evolved differences in these traits^46^. Differences in trait values in a common environment such as this are considered to be due to evolution in those traits, as populations in different experimental treatments were founded from the same stock population^46^.

To conduct growth rate assays, we placed offspring from core, edge and shuffled populations in individual patches at densities of 5, 10, 15 and 20 to reproduce for 24 h. This allowed us to compare growth rates among G1 descendants from the core and edge of structured populations and G1 descendants from shuffled populations and to examine how growth rate varied with density among core, edge and shuffled populations.

To conduct dispersal assays, we placed offspring from core, edge and shuffled populations at densities of 10 and 40 in the first patch of freshly prepared landscapes. Beetles remained in the first patch of these landscapes for 48 h to equilibrate movement behaviour within patches before the plastic dispersal barriers were removed for a 24 h dispersal period. After dispersal finished, the number of beetles that dispersed away from the patch of origin was recorded for each replicate. All of the patches and landscapes for all growth and dispersal assays were kept in the same incubator conditions as previously described.

### Statistical analyses

For each landscape at each generation, we recorded the furthest distance spread, defined as the furthest patch to have a single beetle present (as a single beetle could produce offspring if it was a mated female). To analyse expansion speed, we used a linear mixed effects model with distance spread as the response variable. Fixed effects were generation (a continuous variable allowing estimation of the slope of distance on time, which is the mean expansion speed), treatment (a categorical variable with two levels: structured and shuffled) and the interaction of treatment and generation (that represents the difference in mean expansion speed between structured and shuffled populations). To account for the non-independence of repeated data from the same landscapes, we included a random effects term to allow the speed to vary among replicate landscapes (that is, a random slopes model)^47^. These random slopes were nested within a second random term, block, to account for the three temporal blocks of the experimental design. To test for the significance of treatment on expansion speed, we used a parametric bootstrap to calculate the *P* value for the interaction of treatment and generation. For this and all other bootstrapped *P* values and CIs 95% confidence intervals described below, we performed 10,000 simulations.

For each landscape at each generation, we calculated the standard deviation (s.d.) and CV of distance spread. We used multiple regression to model the linear change of the s.d. and CV through time and to test for a significant effect of treatment or interaction of treatment by generation (likelihood ratio test). This model contained no random effects as each treatment had one summary data point (s.d. or the CV) per generation. Similarly, we used multiple regression to model the relationship of the s.d. to the mean distance spread and to test for a significant interaction of treatment with the mean (likelihood ratio test). Model predictions for the s.d. were transformed to variances for display and comparison with experimental results.

Data from the dispersal assay (number of beetles dispersed away from the origin patch out of the number starting in the origin patch) were analysed using a generalized linear mixed effects model with a binomial distribution and a logit link function for the probability that a beetle disperses away from the origin patch. Fixed effects included population density starting in the origin patch, location of population origin (core, edge or shuffled) and the interaction of density and location. Random intercepts were modelled for each landscape nested within temporal block. To test for a significant effect of treatment or density on dispersal probability, we used a parametric bootstrap to calculate the *P* value for location and the interaction between density and location.

Data from the growth rate assay were analysed using a linearized Ricker model previously developed for this system^48^. As described in Hufbauer *et al*.^48^ the population dynamics of *T. castaneum* in this system can be modelled with a generalized Ricker model allowing for a potential Allee effect: *N*_*t+*1_*=RN*_*t*_^*θ*^ *e*^−*α*^, where *N*_*t*_ is population density (number of beetles per patch) in generation *t*, *R* is the finite growth rate, *α* is the strength of negative density dependence (egg cannibalism by adult beetles) and *θ* is the strength of the Allee effect with a value of 1 corresponding to no Allee effect. This model can be linearized via a log transformation to yield:


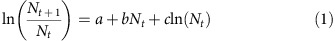


where *a*=ln(*R*), *b*=−*α* and *c*=*θ*−1. We used a mixed effects implementation of this model to fit the data from the growth rate assay. Fixed effects for this model included population density, the natural log of population density, location of population origin, the interaction between location and density (to allow *α* to vary among locations) and the interaction between location and log density (to allow *θ* to vary among locations). Random intercepts for landscapes nested within temporal blocks were included as with the dispersal analysis. We used a parametric bootstrap to test for the significance of location on the intercept of the model (ln(*R*)) as well as the interaction between location and density to test for evolved differences in *α* between offspring from core and edge populations.

To assess correlations between evolved differences in dispersal ability and intrinsic growth rates, we used data from the dispersal and growth rate assays from descendants of the edge populations of structured landscapes. Using landscapes with both a dispersal and growth rate assay, we plotted the proportion of beetles that dispersed away from the origin patch under low-density conditions (from the dispersal assays) against the population growth rate averaged across assay densities (from the growth rate assays). We then calculated Pearson's *r* to assess the correlation between the two.

All statistical analyses were performed using R^49^ (version 3.2.3). Linear mixed effects models and generalized linear mixed effects models were fitted using the lmer and glmer functions in the package lme4 (ref. 50) (version 1.1.11). Bootstraps were performed using the packages pbkrtest^51^ (version 0.4.6) and boot^52^ (version 1.3.18). For all analyses, we checked model assumptions using plots of residuals, quantiles, influence and leverage where appropriate.

### Expected maximum change in expansion speed

To estimate the expected change in expansion speed between the structured and shuffled landscapes, we used the well-known approximation for expansion speed, 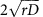, in which *r* is the intrinsic growth rate and *D* is the diffusion coefficient^18^. Using data from the phenotypic assays, we calculated the changes in *r* and *D* between edge populations and shuffled landscapes to determine the expected change in speed. We calculated intrinsic growth rates as *r=*ln(*R*), where *R* was estimated from the data as described above. To calculate the diffusion coefficients, we assumed exponentially distributed waiting times for individual beetles to leave a patch. This assumption means that the probability *p* of an individual leaving a patch over a time period *T* is given by


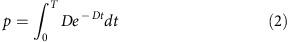


Solving the above integral for *D* and setting *T*=1 (to calculate the diffusion coefficient over a single dispersal period) yields 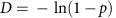, where *p* was estimated from the data as described above. The proportional change in speed is then given by 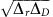, where Δ_*r*_ and Δ_*D*_ are the proportional changes in *r* and *D* between edge populations and shuffled landscapes.

### Data availability

All data used in this study are available from the corresponding author on request.

## Additional information

**How to cite this article:** Weiss-Lehman, C. *et al*. Rapid trait evolution drives increased speed and variance in experimental range expansions. *Nat. Commun.* **8,** 14303 doi: 10.1038/ncomms14303 (2017).

**Publisher's note:** Springer Nature remains neutral with regard to jurisdictional claims in published maps and institutional affiliations.

## Supplementary Material

Supplementary Figures Supplementary Figures 1-4

Peer Review File
