# Supplementary material for: Rapid trait evolution drives increased speed and variance in experimental range expansions
Source: Nat Commun. 2017 Jan 27;8:14303. doi: 10.1038/ncomms14303 (PMC5290145; doi:10.1038/ncomms14303)
Supplement: Supplementary Figures — 1-4 [file ncomms14303-s1.pdf]

***Supplementary Figures***

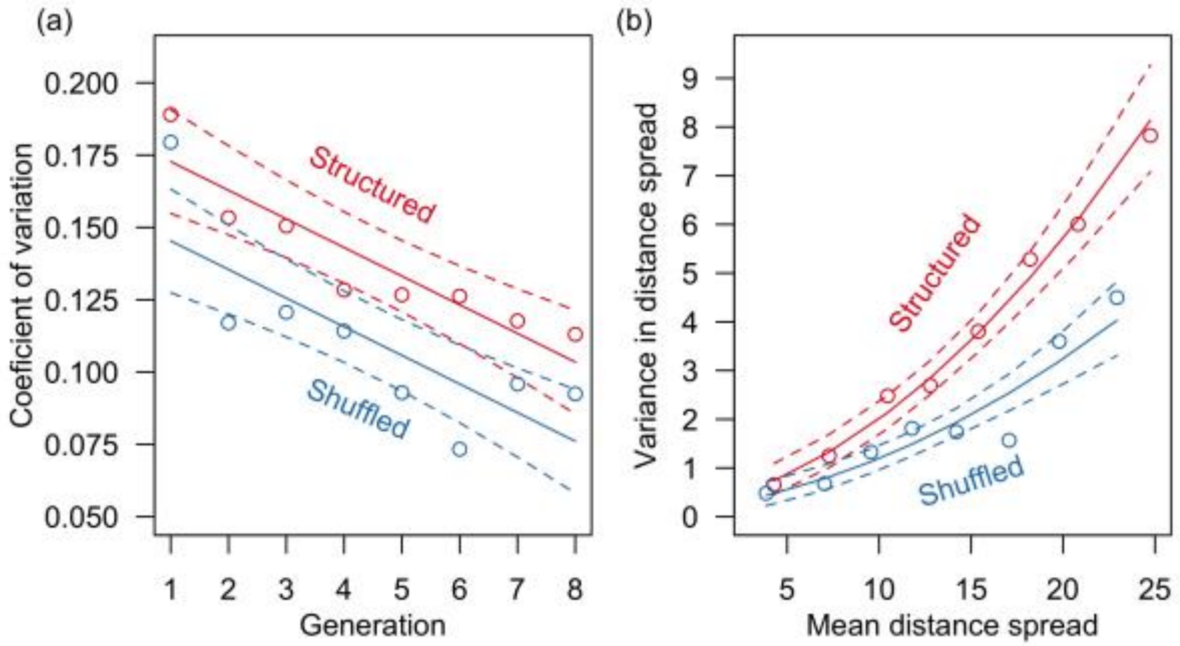


**Supplementary Figure 1 | Increased variation in distance spread in structured populations**

The coefficient of variation (CV) in distance spread through time is shown in (a) and the relationship between the variance in distance spread and the mean distance spread is shown in (b). Solid and dashed lines are means and 95% confidence intervals, respectively, from the regression models described in the methods. Open circles are the observed CV and variance calculated from the replicate populations of the structured (n = 28) and shuffled (n= 29) treatments. The structured treatment is shown in red and the shuffled treatment is shown in blue. Structured populations had a higher coefficient of variation than shuffled populations (likelihood ratio test, treatment effect: p = 0.004) and a significantly different relationship between the variance and the mean compared to shuffled populations (likelihood ratio test, treatment by mean interaction: p = 0.025).

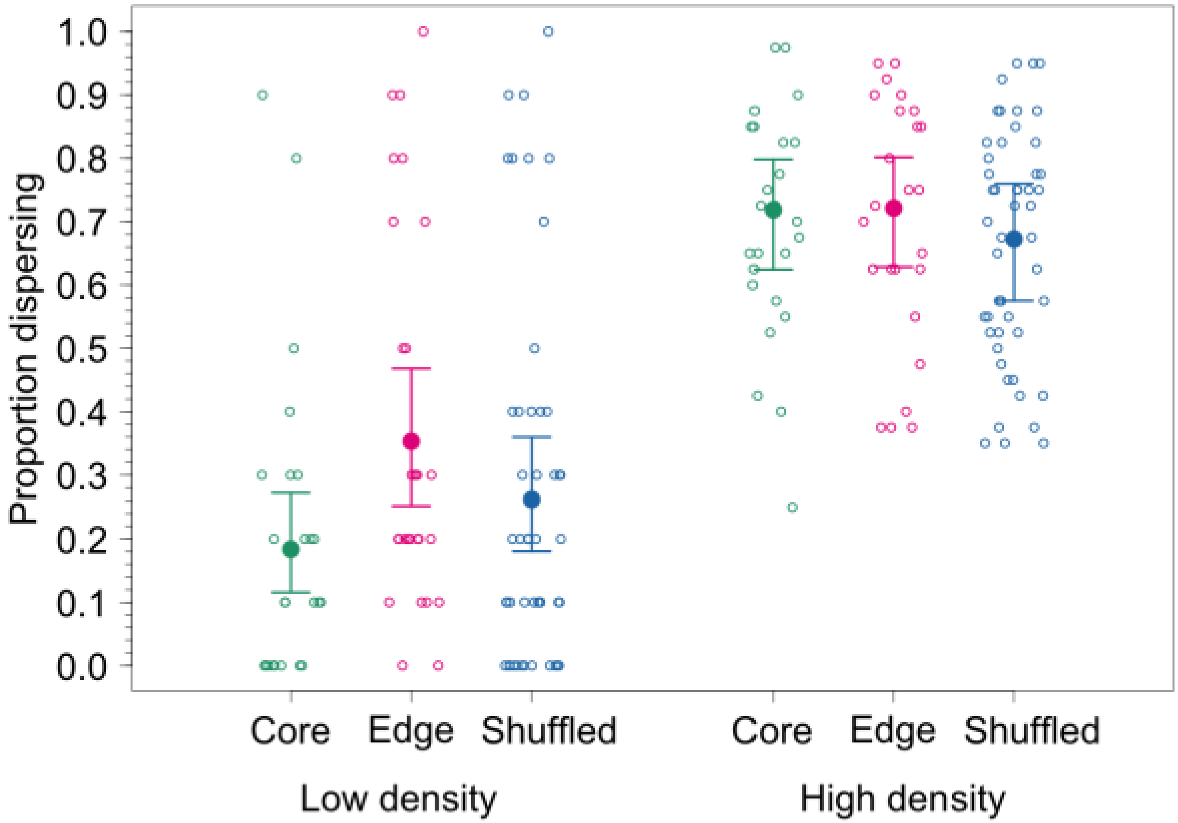


**Supplementary Figure 2 | Evolved density dependence of dispersal in phenotypic tests**

Open circles are the observed proportion of G1 beetles dispersing out of the natal patch in each replicate of each location and density. Replicates for G1 descendants from the core (n = 28) and edge (n = 28) of structured landscapes and G1 descendants from shuffled landscapes (n = 58) are shown. Density treatments were low = 10 and high = 40. Filled circles and error bars are means and 95% confidence intervals from the generalized linear mixed effects model described in the methods. Core populations are shown in green, edge populations in fuchsia, and shuffled populations in blue. Spatial evolution led to a higher proportion of beetles dispersing from edge populations at low density (parametric bootstrap, interaction of density and location: $p = 0.0008$).

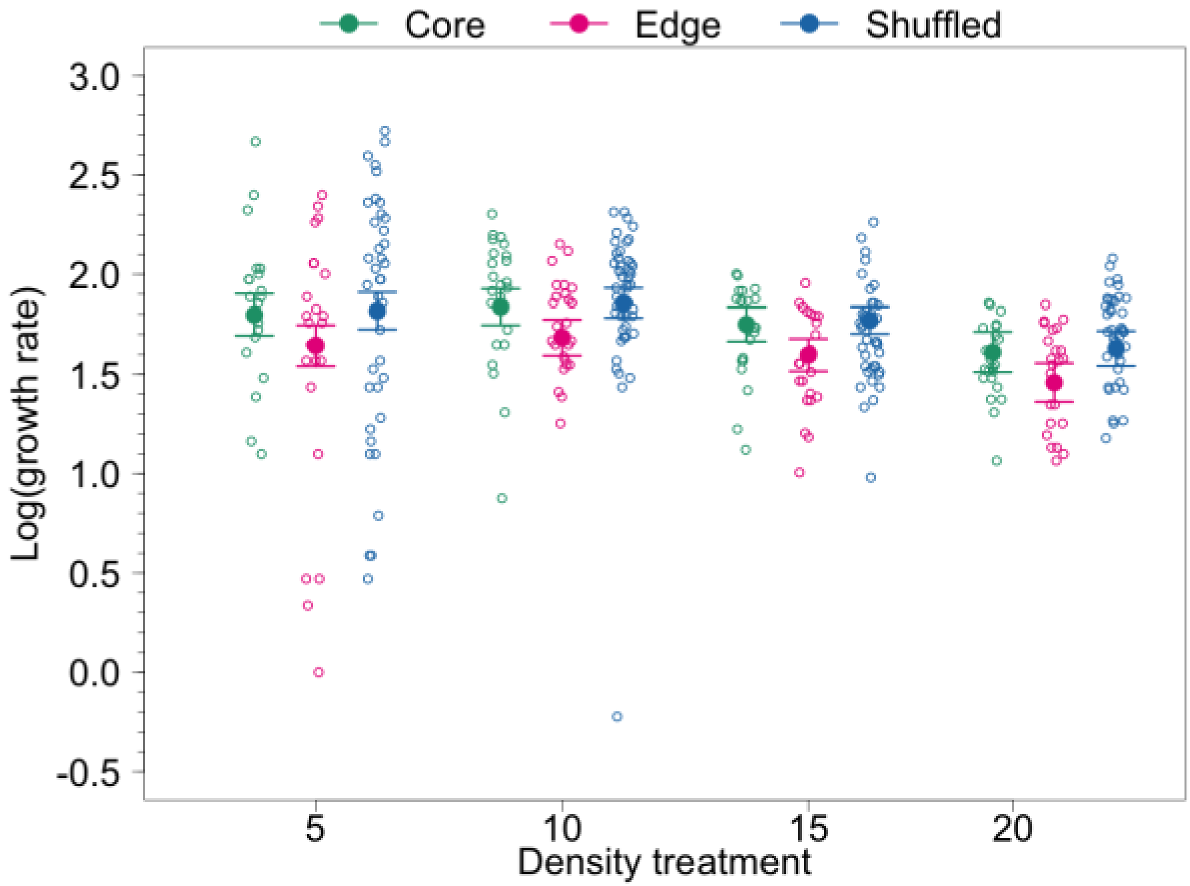


**Supplementary Figure 3 | Negative density dependence of growth rate among locations**

The natural log of growth rate (defined as the ratio of population density at time $t + 1$ to population density at time $t$) for G1 descendants of beetles taken from the core and edge of structured landscapes and G1 descendants from shuffled landscapes. Growth rates were determined at four levels of density. The number of replicates at each of the four density levels respectively were: core, n = 20, 24, 22, 22; edge, n = 23, 27, 23, 23; shuffled, n = 38, 48, 39, 39. Replication differs among locations and densities due to variation in the number of G1 offspring available. Open circles are replicate populations, filled circles are means, and error bars are bootstrapped 95% confidence intervals. Core populations are shown in green, edge populations in fuchsia, and shuffled populations in blue. Spatial evolution resulted in lower intrinsic growth rates in edge populations (parametric bootstrap, effect of location: p = 0.0012), but no difference in the strength of density dependence (parametric bootstrap, interaction of density and location: p = 0.4285).

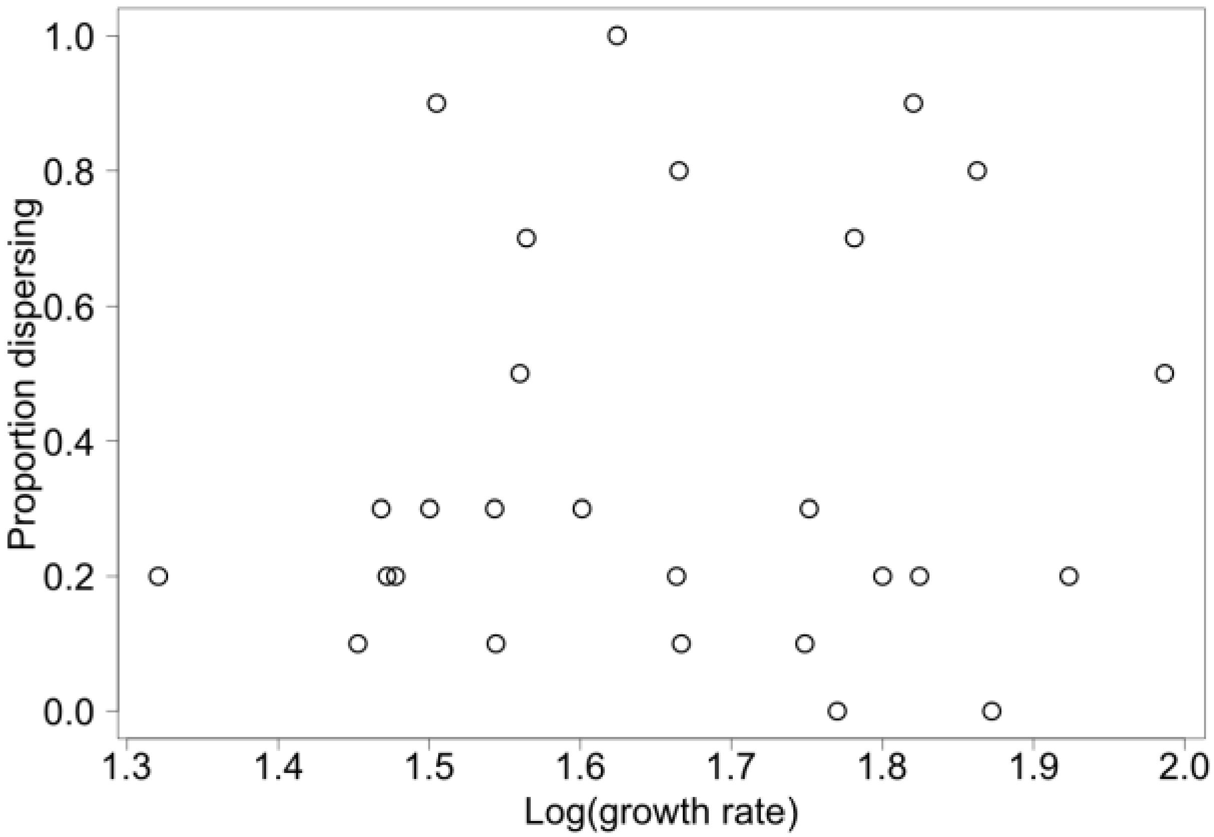


**Supplementary Figure 4 | No correlation of dispersal ability and intrinsic growth rate**

The observed proportion of beetles dispersing in low-density conditions plotted against the natural log of growth rate. Dispersal and growth rate data are from the assays of G1 descendants of edge populations in structured landscapes. Low density conditions for dispersal were defined as 10 adult beetles. Growth rate was defined as the ratio of population density at time $t + 1$ to population density at time $t$. Log(growth rate) was averaged across density levels in the growth assay to provide a relative measure of intrinsic growth rate. The correlation between these two traits was weak and not significantly different from zero (Pearson's correlation ($n = 28$): $r = 0.0785$, $p = 0.6872$).
